# Mutation rate of SARS-CoV-2 and emergence of mutators during experimental evolution

**DOI:** 10.1093/emph/eoac010

**Published:** 2022-03-29

**Authors:** Massimo Amicone, Vítor Borges, Maria João Alves, Joana Isidro, Líbia Zé-Zé, Sílvia Duarte, Luís Vieira, Raquel Guiomar, João Paulo Gomes, Isabel Gordo

**Affiliations:** 1 Instituto Gulbenkian de Ciência, Oeiras, Portugal; 2 Bioinformatics Unit, Department of Infectious Diseases, National Institute of Health Doutor Ricardo Jorge (INSA), Lisbon, Portugal; 3 Centre for Vectors and Infectious Diseases Research, Department of Infectious Diseases, National Institute of Health Doutor Ricardo Jorge (INSA), Lisbon, Portugal; 4 BioISI—Biosystems & Integrative Sciences Institute, Faculty of Sciences, University of Lisbon, Lisbon, Portugal; 5 Innovation and Technology Unit, Department of Human Genetics, National Institute of Health Doutor Ricardo Jorge (INSA), Lisbon, Portugal; 6 Centre for Toxicogenomics and Human Health (ToxOmics), Genetics, Oncology and Human Toxicology, Nova Medical School|Faculdade de Ciências Médicas, Universidade Nova de Lisboa, Lisbon, Portugal; 7 National Reference Laboratory for Influenza and Other Respiratory Viruses, Department of Infectious Diseases, National Institute of Health Doutor Ricardo Jorge (INSA), Lisbon, Portugal

**Keywords:** SARS-CoV-2, experimental evolution, mutation rate, mutator, virus adaptation

## Abstract

**Background and objectives:**

To understand how organisms evolve, it is fundamental to study how mutations emerge and establish. Here, we estimated the rate of mutation accumulation of SARS-CoV-2 *in vitro* and investigated the repeatability of its evolution when facing a new cell type but no immune or drug pressures.

**Methodology:**

We performed experimental evolution with two strains of SARS-CoV-2, one carrying the originally described spike protein (CoV-2-D) and another carrying the D614G mutation that has spread worldwide (CoV-2-G). After 15 passages in Vero cells and whole genome sequencing, we characterized the spectrum and rate of the emerging mutations and looked for evidences of selection across the genomes of both strains.

**Results:**

From the frequencies of the mutations accumulated, and excluding the genes with signals of selection, we estimate a spontaneous mutation rate of 1.3 × 10^*−*6^ ± 0.2 × 10^−6^ per-base per-infection cycle (mean across both lineages of SARS-CoV-2 ± 2SEM). We further show that mutation accumulation is larger in the CoV-2-D lineage and heterogeneous along the genome, consistent with the action of positive selection on the spike protein, which accumulated five times more mutations than the corresponding genomic average. We also observe the emergence of mutators in the CoV-2-G background, likely linked to mutations in the RNA-dependent RNA polymerase and/or in the error-correcting exonuclease protein.

**Conclusions and implications:**

These results provide valuable information on how spontaneous mutations emerge in SARS-CoV-2 and on how selection can shape its genome toward adaptation to new environments.

**Lay Summary:** Each time a virus replicates inside a cell, errors (mutations) occur. Here, via laboratory propagation in cells originally isolated from the kidney epithelium of African green monkeys, we estimated the rate at which the SARS-CoV-2 virus mutates—an important parameter for understanding how it can evolve within and across humans. We also confirm the potential of its Spike protein to adapt to a new environment and report the emergence of mutators—viral populations where mutations occur at a significantly faster rate.

## BACKGROUND AND OBJECTIVES

Mutation is the principal process driving the origin of genetic diversity. The mutation rate is a function of replication fidelity and represents the intrinsic rate at which genetic changes emerge, upon which selection can act. The substitution rate, instead, is a measure of mutation accumulation in a given period of time and embeds the effects of selection [1]. These rates and the spectrum of mutations that emerge and spread are fundamental to our understanding of how organisms evolve and how new variants are purged or established in natural populations.

Laboratory microbial evolution experiments allow to determine how fast mutations accumulate [2, 3], and combining them with high-throughput sequencing is one of the best methods to estimate mutation rates, determine how they vary along the genome [4] and study the extent to which convergent evolution occurs [5, 6].

In DNA-based microbes, the genomic mutation rate per-cell per-generation, estimated in laboratory conditions, is close to a constant, but can span orders of magnitudes when measured per-nucleotide [7]. In RNA viruses, the replication fidelity also varies remarkably [8, 9]. The basic mutation rates, expressed as nucleotide substitutions per-site per-cell infection (s/n/c), range between 10^−6^ and 10^−3^ for the several positive ssRNA viruses which have been studied [10]. Importantly, our current knowledge of the mutation rate of the human beta-coronavirus SARS-CoV-2—cause of the COVID-19 pandemic [11]—is based on estimates from different coronaviruses [10, 12–14]. While there are estimates of the per-site per-year mutation rate of SARS-CoV-2 (∼1.12 × 10^−3^ nt^−1^ year^−1^ [15]), we still lack a direct quantification of the amount of mutations that can be generated in a single infection cycle. Here, via experimental evolution in Vero cells of two natural variants of SARS-CoV-2 [16], followed by whole genome sequencing, we characterized the spectrum of the emerging mutations and estimated their spontaneous mutation rate per-site per-cell infection. We further identified specific targets of selection that occurred during the experimental evolution. Notwithstanding that the rates and the mutations observed here might depend on the specific laboratory conditions (which are far from those in the natural environment of SARS-CoV-2), our study provides important information about the basic biology of this virus and can motivate future researchers to further investigate its adaptive potential and genetic constraints.

## METHODOLOGY

### Virus growth and *in vitro* assay

Vero E6 (African green monkey, *Cercopithecus aethiops* kidney epithelial cells, ATCC^®^ CRL 1586™) cells were cultured at 37°C and 5% CO_2_ in Minimum Essential Medium (MEM 1×, Gibco^®^) supplemented with 10% fetal bovine serum (FBS), penicillin (100 units/ml) and streptomycin (100 µg/ml) + fungizone. The two clinical isolates Portugal/PT0054/2020 and Portugal/PT1136/2020, isolated at the National Institute of Health Doutor Ricardo Jorge (INSA), were used to produce the ancestors of the experimental evolution, CoV-2-D and CoV-2-G, which seeded the two laboratory evolution experiments. For this, the initial SARS-CoV-2 stock was produced by infecting Vero E6 cells (freshly grown for 24 h) and incubating the cells for 72 h. The culture supernatant was stored in aliquots at −80°C. The Median Tissue Culture Infectious Dose (TCID_50_) of viral stock was calculated according to the method of Reed and Muench [17]. All work with infectious SARS-CoV-2 strains was done inside a Class III microbiological safety cabinet in a containment level 3 facility at the Centre for Vectors and Infectious Diseases Research (INSA).

From the stored stocks, two 96-well plates fully inoculated with 50 µl of Vero E6 cells (2.0 × 10^4^ cells) grown for 24 h were infected with 50 µl of the SARS-CoV-2 strains viral suspension (2.0 × 10^3^ viruses) at a multiplicity of infection of 0.1. MEM supplemented with 10% FBS, penicillin (100 units/ml) and streptomycin (100 µg/ml) + fungizone was added to each well (50 µl) and the plates were incubated for 24 h. Each well had a final volume of 150 µl. Every day, for 15 days, serial passages were done by passaging 50 µl of the culture supernatant to 96-well plates (one for each SARS-CoV-2 strain under study) fully inoculated with 50 µl of Vero E6 cells (2.0 × 10^4^ cells) using the same procedure and incubated in the same conditions. At Day 15, total nucleic acids were extracted from 100 µl of viral suspension of each well in each plate (96 samples of Day 15 for each strain) using the automated platform NUCLISENS easyMAG (Biomérieux). Confirmation of nucleic acid integrity and rough concentration estimative was made before sequencing experiment by RT-qPCR of eight random chosen samples from each plate at Day 15 (CoV-2-D and CoV-2-G) using Novel Coronavirus (2019-nCoV) RT-PCR Detection Kit (Fosun Diagnostics). Samples from inoculation suspension (Day 1) were also analyzed. All samples presented values of 7–10 Ct (cycle threshold). When we infect the cells with 2 × 10^3^ plaque-forming units (PFUs), after 24 h, the number of PFUs is around 2 × 10^6^. So, assuming no major fluctuations in the viral load of the transferred suspension throughout the 15 passages and assuming a yield of approximately 1000 PFU/cell [14], the estimated number of replication cycles per passage is around 1 (i.e. (2 × 10^3^) × 10^3^ = 2 × 10^6^).

### SARS-CoV-2 genome sequencing and bioinformatics analysis

Genome sequencing was performed at INSA following an amplicon-based whole-genome amplification strategy using tiled, multiplexed primers [18], according to the ARTIC network protocol (https://artic.network/ncov-2019;https://www.protocols.io/view/ncov-2019-sequencing-protocol-bbmuik6w) with slight modifications, as previously described [16]. Briefly, after cDNA synthesis, whole-genome amplification was performed with NEBNext^®^ Q5^®^ Hot Start HiFi DNA polymerase using two separate pools of tiling primers [Pools 1 and 2; primers version V3 (218 primers) was used for all samples: https://github.com/artic-network/artic-ncov2019/tree/master/primer_schemes/nCoV-2019]. The two pools of multiplexed amplicons were then pooled for each sample, followed by post PCR clean-up and Nextera XT dual-indexed library preparation, according to the manufacturers’ instructions. Sequencing libraries were paired-end sequenced (2 × 150 bp) on an Illumina NextSeq 550 apparatus, as previously described [19]. Sequence read quality analysis and mapping was conducted using the bioinformatics pipeline implemented in INSaFLU (https://insaflu.insa.pt/; https://github.com/INSaFLU; https://insaflu.readthedocs.io/en/latest/; as of 10 March 2021), which is a web-based (and also locally installable) platform for amplicon-based next-generation sequencing data analysis [19]. We performed raw reads quality analysis using FastQC v0.11.9 (https://www.bioinformatics.babraham.ac.uk/projects/fastqc), followed by quality improvement using Trimmomatic v.0.27 (http://www.usadellab.org/cms/index.php?page=trimmomatic; HEADCROP:30 CROP:90 SLIDINGWINDOW:5:20 LEADING:3 TRAILING:3 MINLEN:35 TOPHRED33), with reads being conservatively cropped 30 bp at both ends for primer clipping. Reference-based mapping was performed against the Wuhan-Hu-1/2019 reference genome sequence (https://www.ncbi.nlm.nih.gov/nuccore/MN908947.3; NC_045512.2) using the Burrow-Wheeler Aligner (BWA_MEM) v.0.7.12 (r1039) (http://bio-bwa.sourceforge.net/) [20] integrated in multisoftware tool Snippy (https://github.com/tseemann/snippy) available in INSaFLU. The obtained median depth of coverage throughout the genome for CoV-2-D and CoV-2-G samples (except two samples excluded due to low coverage) was 4807 (IQR = 3969-5242) and 5154 (IQR = 4802-5439), respectively. Variant (SNP/indels) calling was performed over BAM files using LoFreq v.2.1.5 (*call* mode, including *–call-indels*) [21], with indel qualities being assessed using Dindel [22]. Mutation frequency analysis was dynamic and contingent on the depth of coverage of each processed site, e.g. minor mutations at ‘allele’ frequencies of 10%, 2% and 1% (minimum cut-off used) were validated for sites with depth of coverage of at least 100-fold, 500-fold and 1000-fold, respectively. The median depth coverage per site for all validated mutations in CoV-2-D and CoV-2-G samples was 4219 (IQR = 2508-6649) and 6424 (IQR = 3076-10104), respectively. In order to assess if proximal SNPs and/or indels belong to the same mutational event (and thus, avoid overestimating the mutation rate), we identified all consecutive mutations separated by ≤12 bp. The mutations more likely to represent a single mutation event, i.e., those with similar frequencies (differing by ≤ 2.5%), were further visually inspected using IGV (http://software.broadinstitute.org/software/igv/) to confirm/exclude their co-localization in the same reads. In total, this curation led to the identification 37 SNPs/indels that were collapsed into 13 complex or multi-nucleotide polymorphisms (MNP). The effect of mutations on genes and predicted protein sequences was determined using Ensembl Variant Effect Predictor (VEP) version 103.1 (https://github.com/Ensembl/ensembl-vep; available as a self-contained Docker image) [23]. To obtain a refined annotation including all ORF1ab sub-peptides, the GFF3 genome annotation file (relative to the reference Wuhan-Hu-1/2019 genome of SARS-CoV-2, acc. no. NC_045512.2) available in the coronapp COVID-19 genome annotator (http://giorgilab.unibo.it/coronannotator/) [24] was adapted to generate an annotation GTF file for input for the *–gtf* parameter. The parameter *–distance* was set to 0. Supplementary Table S1 summarizes all mutations detected in this study and their distribution across clinical, ancestral cultures and end-point cultured lines (15th passage). SARS-CoV-2 consensus sequences obtained directly from clinical samples for CoV-2-D (Portugal/PT0054/2020) and CoV-2-G (Portugal/PT1136/2020) viruses are available in GISAID under the accession numbers EPI_ISL_421457 and EPI_ISL_511683, respectively. Reads generated at the end of the experimental evolution study were deposited in the European Nucleotide Archive (ENA) (https://www.ebi.ac.uk/ena/data/view/PRJEB43731).

### Simulations of the neutral mutation accumulation

To obtain a non-equilibrium neutral expectation of the site frequency spectrum of mutations, we performed forward-simulations to model mutation accumulation using the mutation rate inferred from the experiment. We model an organism with a bi-allelic genome of size *L* = 30 000 (∼SARS-CoV-2). An initially isogenic population undergoes 15 cycles of growth, mutation and bottleneck, according to the following life cycle:

1. A clonal population starts with an inoculum size of 2000.
2. Each genome replicates × times. We assume the burst size × to be Poisson distributed with mean 1000.
3. For each of the replicating genomes, we introduce a Poisson number of mutations with mean 0.1 (corresponding to a rate of 3.3 × 10^−6^ nt^−1^ cycle^−1^). We assume mutations to emerge with uniform probability in the parental genome and we allow for back-mutation.
4. After replication and mutation, we sample 1/1000 of the individual genomes.
5. Repeat steps 2–4, 15 times.

We validated the simulation code by confirming expected outcomes: mutations accumulate linearly over time and the posterior estimation of the mutation rate retrieves the original value (bottom distribution in Supplementary Fig. S3b).

After 15 cycles, we collect the artificial genomes from 100 independent simulations, and compute their site frequency spectrum as in the experiment.

In order to test whether cross-well contamination could justify the observed site frequency distribution, we modified the previous algorithm by introducing migration. At each Cycle *t*, after each bottleneck event, a fraction of viral genomes (*m* = 0.1) is replaced by migrants sampled from a pool of genomes that have undergone *t* cycles of growth.

In order to test whether a more heavy-tailed distribution of burst size could explain the observed site frequency distribution, we modified the previous algorithm by assuming a log-normal burst size distribution ($x=e^{z},z∼\mathrm{Normal}(\mu ≅6.4,\sigma =1)$), such that its mean $\left(\mathrm{mean}\left(x\right)=e^{\mu +\frac{s^{2}}{2}}=1000\right)$ is equivalent to the previously assumed Poisson distribution. The algorithm was written in R (version 3.6.1) and the results analyzed in RStudio [25].

### Mutation accumulation rates in all, synonymous and non-synonymous sites

To quantify the rate at which mutations accumulate during the experiment, we compute $M_{a}(r)=\frac{\sum f_{r}}{P*L_{r}}$, where $f_{r}$is the frequency of all mutations observed in region r, *P* = 15 is the number of passages and $L_{r}$ is the length of region r. For the genome-wise mutation accumulation $L_{r}=29903$, the entire genome of SARS-CoV-2. We also computed the mutation accumulation rates at synonymous and non-synonymous sites. In these cases, the synonymous rate is given by $M_{a}(r,\mathrm{syn})=\frac{\sum f_{r}}{P*L_{r}*p_{r,\mathrm{syn}}}$, where $p_{r,\mathrm{syn}}$ is the proportion of mutations in region r, leading to synonymous changes. Equivalently the non-synonymous rate is $M_{a}(r,n\_\mathrm{syn})=\frac{\sum f_{r}}{P*L_{r}*p_{r,n\_\mathrm{syn}}}$. In practice, assume the region of interest has sequence r: ATGTTT. For each base, we count the proportion of mutations that would change (or not) the corresponding amino acid. In the example $p_{r,n\_\mathrm{syn}}=3/3+3/3+3/3+3/3+3/3+2/3=17/18$, 17 mutations out of the possible 18 are non-synonymous and only one is synonymous (ATGTTc). Therefore, in this example, the total size is $L_{r}=6$, $p_{r,n\_\mathrm{syn}}=17/18$ and $p_{r,\mathrm{syn}}=1/18$. Following this method, we calculated the genome-wise and gene-specific mutation accumulation rates in all, synonymous or non-synonymous sites, reported throughout the entire manuscript. The genomic sequences of each region were retrieved from NCBI (entry: NC_045512).

### pN/pS calculation and confidence interval

To test for the action of selection, we estimated the ratio of non-synonymous to synonymous polymorphism rates (*pN/pS*) [26] for each of the two lineages. Within a given region *r*, we computed *pN(r)* as the summed frequencies of all the observed non-synonymous mutations over the number of all possible non-synonymous changes in that region: $pN\left(r\right)=\frac{\sum f_{r}}{N_{r,n\_\mathrm{syn}}}$, where $N_{r,\mathrm{syn}}={3L}_{r}p_{r,n_{\mathrm{syn}}}$. Equivalently, we computed the synonymous counterpart: $pS\left(r\right)=\frac{\sum f_{r}}{N_{r,\mathrm{syn}}}$. In the previous example, within the region r: ATGTTT, $N_{r,n\_\mathrm{syn}}=17$ while $N_{r,\mathrm{syn}}=1$. Finally, the *pN/pS* statistics is the ratio of pN and pS and its expected value is 1 under neutrality. We report the *pN/pS* together with its bootstrap-based confidence interval. In particular, we report the 95% percentiles of the *pN/pS* distribution obtained by resampling the observed synonymous and non-synonymous mutations 1000 times. As the *pN/pS* is a ratio of proportions, to test its deviation from 1 (or equivalently pN≠pS), we perform two proportions z-tests and correct for multiple comparisons using the Benjamini–Hochberg method.

### Convergence statistics

To test for the significance of the observed convergent evolution, we compared the average pairwise nucleotide distance (π) within and between groups. Considering the SNPs accumulated in each background we compute $\pi_{\mathrm{within}}≔\frac{\sum_{\left(i,j\right)}^{N}d(i,j)}{\left(\begin{array}{c} 2\\ N \end{array}\right)}$, where N is the set of evolved lines within each background (*N* = 96 for CoV-2-D and 79 for CoV-2-G) and d(i,j) is the number of mutations that differ between lines *i* and *j.*

$\pi_{\mathrm{between}}$ is then compared as $\frac{\sum_{\left(i,j\right)}^{N1,N2}d(i,j)}{N1\cdot N2}$, where d(i,j) is between the lines of CoV-2-D (N1) and CoV-2-G (N2). Note that, $\pi_{\mathrm{between}}>\pi_{\mathrm{within}}$ might result from background-dependent evolution but also from migration (cross-contamination between lines of the same background) and/or undetected standing variation.

We tested the significance of the amino acid convergence between the spike protein of both backgrounds via the hypergeometric test, where the set of mutated amino acids of CoV-2-D is the number of draws (*n*), the convergent ones are the successes (*k*), the number of amino acids of the spike protein is the total population (*N*), and the set of mutated amino acids of CoV-2-G is the number of possible successes (*K*).

## RESULTS

### Experimental evolution design and ancestor backgrounds

**Figure 1.**
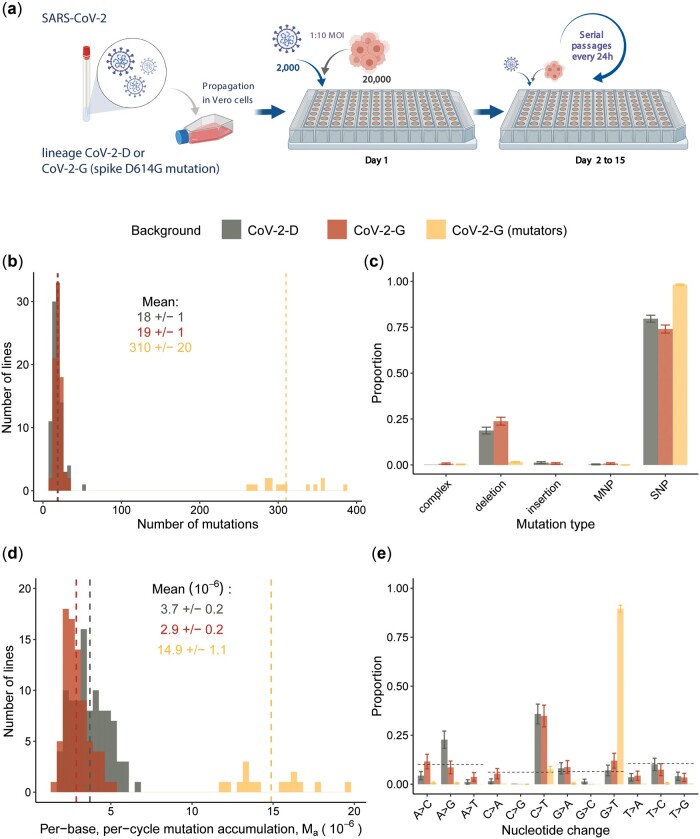
Experimental design and mutation accumulation after 15 passages of SARS-CoV-2 evolution. (**a**) Schematic of the experimental design of the mutation accumulation experiments where two viral backgrounds were propagated in Vero cells (figure created with BioRender.com). (**b**) Number of mutations observed in each well and group; 15 lines of the CoV-2-G background accumulated a larger number of mutations and thus were defined as mutators (gold). The means of each group are presented by vertical dashed lines and reported in the figure (± 2 SEM). (**c**) Proportion of mutation types in each group. Complex mutations and multi-nucleotide polymorphisms (MNP) are defined in the Methodology section. (**d**) Mutation accumulation per base per infection cycle (*M*_a_) was calculated by summing the observed mutation frequencies as: $M_{a}=\frac{\sum f}{P*G}$, where *P* is the number of passages (*P* = 15) and G is the SARS-CoV-2 genome length (G = 29 903). The means of each group are presented by vertical dashed lines and reported in the figure (±2 SEM). (**e**) Proportion of observed nucleotide changes. Dashed lines indicate the expectation given the genome composition under equal mutation probability for each type of nucleotide change. Vertical bars in panels (c) and (e) represent the 95% confidence interval computed as $p\pm z\sqrt{\frac{p(1-p)}{N}},\;z=1.96$

Two SARS-CoV-2 viral strains were isolated from two non-related patients for continuous propagation in Vero cells (see Methodology, Fig. 1a). These were chosen according to their polymorphism at amino acid position 614 of the spike protein: CoV-2-D carries a D and CoV-2-G carries a derived mutation which changes the D into a G. This D614G mutation in the spike protein emerged early in the pandemic, increased the infectivity of the virus and became prevalent worldwide [27]. Here, we want to test for differences in their mutation rates, spectrum and/or in the selective forces as the strains are propagated in cells.

In order to discriminate *de novo* mutations from standing genetic variation, we identified the mutations (relative to the Wuhan-Hu-1/2019 reference genome sequence, Wu et al., 2020) that were already present at the start of our evolution experiment (see the list and their frequencies in Supplementary Fig. S1).

If the mutation rate is similar to that of the mouse hepatitis virus or that of the SARS-CoV (about 3.5 × 10^−6^ and 2.5 × 10^−6^ nt^−1^ cycle^−1^, respectively) [10, 12] hundreds of mutations should accumulate, many of which are expected to be neutral but some could reflect adaptation to the specific conditions used in this experiment.

### Mutation accumulation and spectrum after 15 passages of SARS-CoV-2 evolution

We considered *de novo* mutations those that reached a frequency of at least 1%, supported by a minimum of 10 reads and that were not detected in either the ancestor or the original clinical isolate from which the ancestor was derived (full list in Supplementary Table S1). Propagation of the 96 CoV-2-D-derived lines resulted in 1753 *de novo* mutations, while the 96 lines derived from CoV-2-G resulted in 6181 *de novo* mutations (*n* = 94 as in two lines, the sequencing had poor coverage) (Fig. 1b). The much higher number of mutations in the CoV-2-G background, compared to CoV-2-D, is explained by 15 of these lines where many more mutations were observed (Fig. 1b). These lines, hereafter referred to as mutators, are characterized by a larger proportion of SNPs compared to the non-mutator lines where, instead, deletions account for more than 20% of all *de novo* mutations (Fig. 1c).

The frequency of mutator clones was estimated to be between 1 and 2% after 15 infection cycles, since these were the frequencies measured for the vast majority of mutations observed in the mutator lines. The genetic cause of the mutator phenotype is difficult to determine but it could likely be hidden within the mutations that occurred in the RNA-dependent RNA polymerase (Nsp12) and/or in the error-correcting exonuclease protein (Nsp14) [13]. Indeed, looking at the mutations that are specific to the lineages with mutators, we found eight non-synonymous mutations in Nsp12 (one leading to a stop at amino acid 670) and nine non-synonymous mutations in Nsp14 (one leading to a stop at amino acid 78) (Supplementary Tables S2 and S3). Any of these mutations could potentially lead to the observed change in mutation rate, but none of these has been associated with an increased mutational load of the circulating viruses [28].

Next, we obtained the per-base per-passage rate at which mutations accumulated (M_a_), from the frequencies of the observed mutations. As a 24-h passage in our experiment corresponds to ∼1 cell replication cycle (see Methodology), we hereafter report such rate of mutation accumulation per unit of replication cycle (nt^−1^ cycle^−1^). Interestingly, the non-mutator lines of CoV-2-G show a significantly lower accumulation rate compared to the CoV-2-D lines (*P* < 10^−5^, non-parametric Wilcoxon test) (Fig. 1d). However, this difference between the two backgrounds is more likely due to differences in selection rather than differences in mutation rates, as we will explain later on.

The SNPs accumulated over 15 passages show that both genomic backgrounds have a strong propensity to accumulate C->T mutations (Fig. 1e), a well-known bias of SARS-CoV-2 [29]. In the mutator lines, the main mutation bias changed from C->T to G->T (Fig. 1e), also observed in SARS-CoV-2 samples collected during the COVID-19 pandemic [30, 31].

It is important to notice that, both the accumulation of mutations and the biases we observe in the data (Fig. 1b–e) might have been shaped by selection and deviate from the neutral rate and spectrum of mutations. In fact, on one hand positive selection can increase the frequencies of beneficial mutations and on the other hand purifying selection can purge the deleterious alleles. Therefore, we next looked for evidences of selection in the mutation accumulation data.

### Signs of selection: site frequency spectrum and heterogeneity across genes

In serial propagation experiments with SARS-CoV-2, it is extremely difficult to transfer a single virus [32]. In our experiment, the effective population size is at least 2000 (see Methodology), and thus unlikely to eliminate the effects of either positive or negative selection [2]. Indeed, several patterns in the data indicate that selection played a significant role in the experimentally evolved SARS-CoV-2 lines.

**Figure 2.**
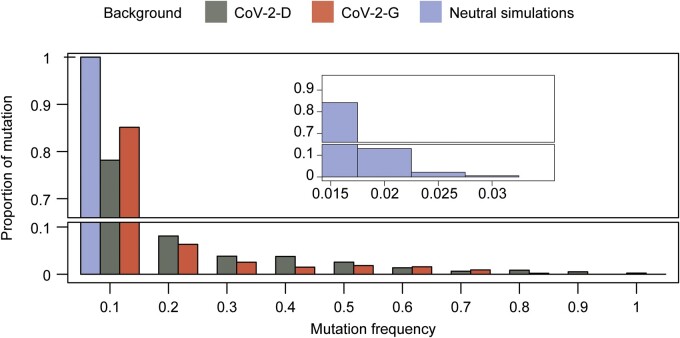
Site frequency spectrum. Proportion of mutations with a given frequency after 15 cycles of propagation in the CoV-2-D (*n* = 96) and CoV-2-G (*n* = 79) genetic backgrounds or under a simulated neutral model of mutation accumulation (*n* = 100, see Methodology). The bump observed at high frequencies in the data is not compatible with the expectation of the neutral model. Here, for the neutral model, we assumed 15 cycles of growth (Poisson-distributed burst size), mutations and bottlenecks without selection (see the effects of larger variation in the viral burst size and of selection in Supplementary Figs S3c and d and S9, respectively)

The distribution of allele frequencies in a sample, i.e. the site frequency spectrum, has a well-known theoretical expectation under a simple equilibrium neutral model of molecular evolution (Chap. 5, p. 233 of B. Charlesworth and D. Charlesworth, 2010 [33]). But, this distribution is sensitive to the action of selection and also to complex demographic events, such as population bottlenecks. Given the bottlenecks occurring in our experiments and the slow evolutionary time elapsed during the 15 infection cycles, the neutral theoretical expectation at equilibrium may not apply. To obtain a non-equilibrium expectation of the site frequency spectrum, we performed forward-simulations (see Methodology). We assumed that neutral mutations occur at a rate of 3.3 × 10^−6^ nt^−1^ cycle^−1^, similar to that estimated from the data, and simulated populations evolving under neutrality. The site frequency spectrum of the mutations accumulated in both CoV-2-D or CoV-2-G lines deviates significantly from the neutral expectation predicted by the simulations (Fig. 2). High frequency mutations are not expected under this neutral model (mutations with frequencies above 30% are reported in Supplementary Fig. S2). To test whether possible contamination among wells could explain the observed site frequency spectrum, we performed additional simulations with migration (see Methodology). Even when considering a migration rate of 10%, the neutral site frequency spectrum is still incompatible with the experimental data (Supplementary Fig. S3a), and the estimation of mutation rate should not be affected (Supplementary Fig. S3b). We also tested whether larger variation in viral burst sizes could generate patterns more similar to the observed (see Methodology). The simulations demonstrate that a more heavy-tailed distribution of burst size can lead some mutations to higher frequencies (compared to the previously assumed Poisson distribution, see Supplementary Fig. S3c) and suggest that such variation in the viral burst size might contribute, together with positive selection, to skew the site frequency spectrum and explain the observed long tail (Fig. 2, Supplementary Fig. S3d).

**Figure 3.**
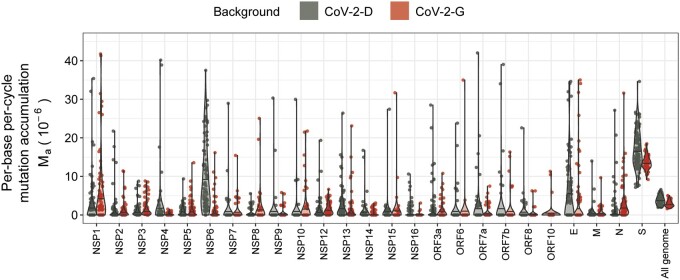
Heterogeneity of mutation accumulation across genes. Per-base mutation accumulation (*M*_a_) computed for each gene and for the entire genome shows heterogeneity. The spike gene has the largest accumulation rate in both backgrounds ($M_{a}^{\left(S\right)}=17.1\;\pm \;1.0,\;13.5\;\pm 0.4\;{\cdot 10}^{-6}$, for the CoV-2-D and CoV-2-G respectively), which is more than four times their genomic average. For resolution purposes, few outliers with *M*_a_ above 45 are not shown (see full set in Supplementary Fig. S4)

A second evidence of selection comes from the considerable variation in the rate of mutation accumulation observed across the SARS-CoV-2 genome (Fig. 3, Supplementary Fig. S4). When excluding the mutator lines, the S gene, which codes for the spike protein, has the highest rate of mutation accumulation among the different genes (Fig. 3). Remarkably, the spike accumulated 13.5 ± 0.4 × 10^−6^ nt^−1^/cycle^−1^ mutations in the CoV-2-G genotype (excluding mutators), and 17.1 ± 1.0 × 10^−6^ in the CoV-2-D genotype, about 5-fold the corresponding genomic averages, suggesting the strong action of positive selection. In the mutator lines, the spike gene accumulated ∼2 times more mutations than in the non-mutators (Supplementary Fig. S4). This observation is in contrast with the 5-fold increase in mutation accumulation across the entire genome (Fig. 1d) and suggests that more complex selective forces might be acting on the mutator phenotype (see the heterogeneity of the mutation rate across the CoV-2-G mutator genome in Supplementary Fig. S4). Overall, the data confirmed that selection has shaped the way mutations accumulated. Therefore, in order to obtain a more accurate quantification of the spontaneous rate of mutation, we performed a more systematic analysis of the sites under selection.

### Identifying regions under selection

From the frequencies of all mutations observed in the CoV-2-D and CoV-2-G non-mutator lines, we computed an accumulation rate of 3.7 × 10^−6^ and 2.9 × 10^−6^ nt^−1^ cycle^−1^, respectively (Fig. 1d). Given that during our experiment, selection affected the allele frequencies (Figs 2 and 3), such rates may deviate from the spontaneous mutation rates of the virus. In order to attempt to estimate the spontaneous mutation rate, we first focused on synonymous mutations, which, if neutral and not linked with sites under selection, should accumulate at the rate at which they occur [34]. Focusing on the synonymous changes, we estimated a basic mutation rate of 3.8 × 10^−6^ nt^−1^ cycle^−1^ for the CoV-2-D background and 1.2 × 10^−6^ nt^−1^ cycle^−1^ for the CoV-2-G (Supplementary Fig. S5a). However, the rate of non-synonymous mutation in CoV-2-D is lower than the synonymous one (Supplementary Fig. S5a and b), suggesting the action of purifying selection on non-synonymous sites or positive selection on the synonymous sites, leading to their increase in frequency [35, 36]. To distinguish between the two cases, we compared the accumulation rate of synonymous mutations in the entire genome ($M_{a}^{\mathrm{Syn}}$), with the accumulation rate of synonymous mutations excluding one gene at a time ($M_{a}^{\mathrm{Syn},\Delta g}$), similar to the Cook’s D approach [37]. This approach revealed that a remarkable accumulation of synonymous mutations in the *Nsp6* gene led to the overestimation of the mutation rate in the CoV-2-D background (Supplementary Fig. S6). In contrast, for the CoV-2-G background, this approach indicates that the estimation of $M_{a}^{\mathrm{Syn}}$=1.2 × 10^−6^ nt^−1^ cycle^−1^ is homogeneous across the genome and can provide a first estimation of its mutation rate (Supplementary Fig. S6b).

**Figure 4.**
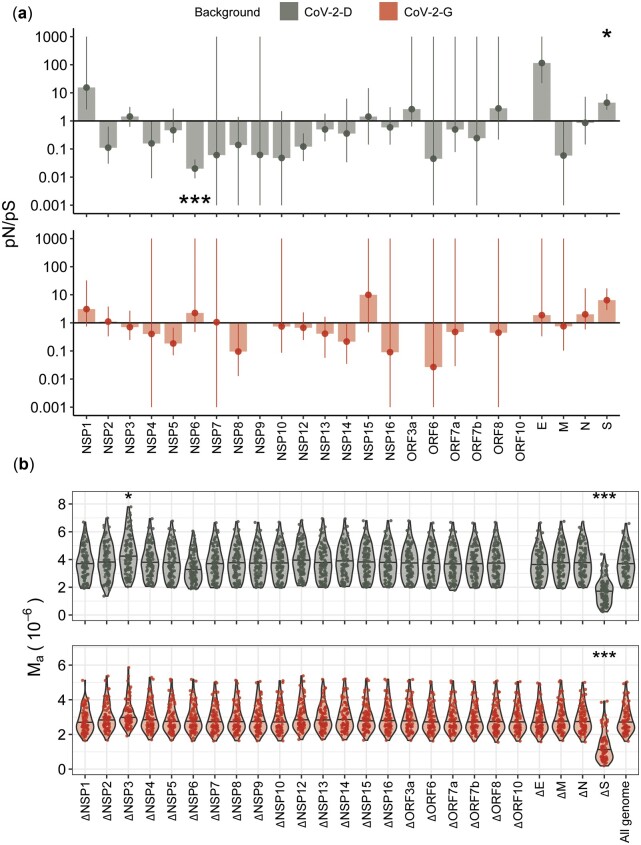
Gene-wise signs of selection. (**a**) The relative proportion of non-synonymous to synonymous polymorphism, *pN/pS*, was computed for each gene and genetic background (see Methodology). The horizontal line indicates the expectation under neutrality (*pN*/*pS=1*), values above suggest positive selection while values below suggest purifying selection. Vertical bars show the 95% distribution of bootstrapped resampling (*n* = 1000) and the stars indicate the genes where *pN/pS* significantly differs from *1* (two proportions z-test, *P*-value < 0.05 (*), 0.01 (**) or 0.001 (***), after Benjamini–Hochberg correction). For the sake of resolution, we show the confidence intervals within the [$10^{-3},\;10^{3}$] range. (**b**) Identifying the genes that affect the estimation of mutation rate. Per-base mutation accumulation (*M*_a_) was computed for the entire genome or by excluding each gene on at the time (e.g. ΔS). The stars indicate the cases where removing the gene leads to an estimation of *M*_a_ significantly different from the all genome (non-parametric Wilcox test, *P*-value < 0.05 (*), 0.01 (**) or 0.001 (***), after Benjamini–Hochberg correction)

To test the robustness of the former estimations of mutation rate, we followed a second approach: identify the regions under selection in either the CoV-2-D or CoV-2-G lines and exclude them from the estimation of the spontaneous mutation rate. First, we compared the relative accumulation of non-synonymous and synonymous mutations, via the *pN/pS* statistics (equivalent of *dN/dS* for polymorphic samples, see Methodology). In the CoV-2-D background, the *pN/pS* of the *S* and the *Nsp6* genes significantly differ from 1 (Fig. 4a, *P*-value = 0.03 and 2 × 10^−13^, respectively, after two proportions z-test and Benjamini–Hochberg correction). The spike accumulated more non-synonymous mutations consistent with the action of positive selection (*pN/pS = 4.44*, 95% confidence interval: [2.47–9.09]), while the Nsp6 accumulated more synonymous mutations, consistent with our previous findings (*pN/pS = 0.02, 95%* confidence interval: [0.01–0.04], Supplementary Fig. S6a). In particular, the synonymous change A11041G was found in 88 evolved populations (out of 96), but also at frequency below our 1% threshold in the ancestral population, suggesting that such mutation was incorrectly considered as *de novo* and that the estimated mutation accumulation in Nsp6 was the resulting artifact.

Due to the limited number of mutations within each gene and the fact that we are comparing evolving populations (rather than divergent species), the *pN/pS* may lack the power to identify additional regions under selection [38]. To overcome this issue and to identify additional genes affecting the estimation of the mutation rate, we computed the rate of mutation accumulation excluding one gene at a time and compared this with the entire genome (similar to what was done before for synonymous mutations only; Supplementary Fig. S6). With this outlier-detecting method, we confirmed that the S gene biased the estimation of mutation accumulation and was likely under positive selection in both CoV-2-D and CoV-2-G backgrounds (Fig. 4b). We also spotted the Nsp3 gene, a genomic region that accumulated significantly fewer mutations than the genomic average, suggesting the action of purifying selection (Fig. 4b).

Overall, we conclude that during our experiment, the spike protein was under strong selection in both backgrounds and additional mutations in different genomic regions have also increased in frequency, possibly due to selection but also through hitchhiking with beneficial mutations.

### Estimation of mutation rates and bias excluding genes with signs of selection

**Figure 5.**
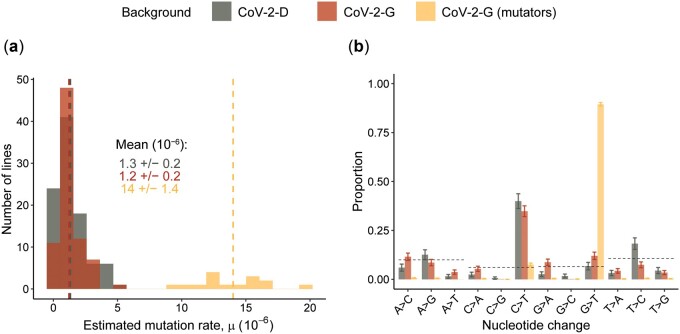
Estimation of mutation rates and bias excluding outlier genes. (**a**) The per-base per-infection cycle mutation rate was calculated by summing the observed mutation frequencies as: $\mu =\frac{\sum f}{P*G}$, where *P* is the number of passages (*P* = 15) and G is the length of SARS-CoV-2 genome excluding the Nsp3, Nsp6 and Spike genes (29903-5835-870-3822 = 19376). The means of each group are presented as vertical dashed lines and reported in the figure (± 2 SEM). (**b**) Proportion of nucleotide changes observed excluding the Nsp3, Nsp6 and Spike genes. Dotted lines indicate the expectation given the genome composition under equal mutation probability for each type of nucleotide change. Vertical bars represent the 95% confidence interval computed as $p\pm z\sqrt{\frac{p(1-p)}{N}},\;z=1.96$

Non-neutral processes have shaped the allele dynamics in our experiment. We identified the Nsp3, Nsp6 and S genes, which have shown signs of selection and/or biased the estimation of mutation accumulation in at least one of the two backgrounds (Fig. 4). Excluding these genes from the analysis allows us to remove, at least in part, high frequency mutations (see Supplementary Fig. S7) and to obtain a more realistic estimate of the SARS-CoV-2 mutation rate prior to selection. By doing so, we estimate a spontaneous mutation rate of 1.3 ± 0.2 × 10^−6^ nt^−1^ cycle^−1^ for the CoV-2-D background, and 1.2 ± 0.2 × 10^−6^ nt^−1^ cycle^−1^ for the CoV-2-G (excluding mutators) (Fig. 5a). The mean estimated mutation rate does not differ between backgrounds (*P* = 0.6, non-parametric Wilcoxon test), suggesting that the previously observed differences were due to selection (Fig. 1d and Supplementary Fig. S5). Importantly, the estimated mutation rate of CoV-2-D and CoV-2-G are also in agreement with those obtained from the synonymous mutations (see Supplementary Fig. S8). Assuming one infection cycle per passage (15 cycles in total), the average mutation rate across backgrounds is 1.3 × 10^−6^ ± 0.2 × 10^−6^ nt^−1^ cycle^−1^ (mean ± 2SEM). Considering variation in the number of cycles occurred in our experiment (i.e. 15 ± 5) broadens the interval of confidence to 1.0 × 10^−6^ to 1.9 × 10^−6^.

Excluding the Nsp3, Nsp6 and S genes was our best attempt at quantifying the spontaneous mutation rate of SARS-CoV-2. It is important to note that, even after removing the regions under selection, neutral mutations might still be found at high frequencies, possibly due to variation in the viral burst size (as discussed earlier, see Supplementary Fig. S3c and d) and/or due to hitchhiking with beneficial mutations. However, in our analysis, after removing the S, Nsp3 and Nsp6 genes, the fraction of mutations with high frequency is largely reduced (see Supplementary Fig. S7) and the few left should not impact the overall estimation of mutation rate (see a simulated example of gene-specific selection and hitchhiking in Supplementary Fig. S9). In contrast, our estimation should still underestimate the real mutation rate due to the fact that we ignored mutations with a frequency below the 1% threshold. Our simulations suggest that the ignored mutations should increase the estimation by < 3 times (Supplementary Fig. S10), finally placing the estimated mutation rate of SARS-CoV-2 between 1 and 5 × 10^−6^ nt^−1^ cycle^−1^.

Finally, we quantified again the relative proportion of single nucleotide changes and confirmed that both backgrounds have a spontaneous bias toward C>T mutations and the mutator changes this bias toward G>T mutations (Fig. 5b), suggesting that such biases are not a consequence of selection.

### Convergent targets of selection on spike

**Figure 6.**
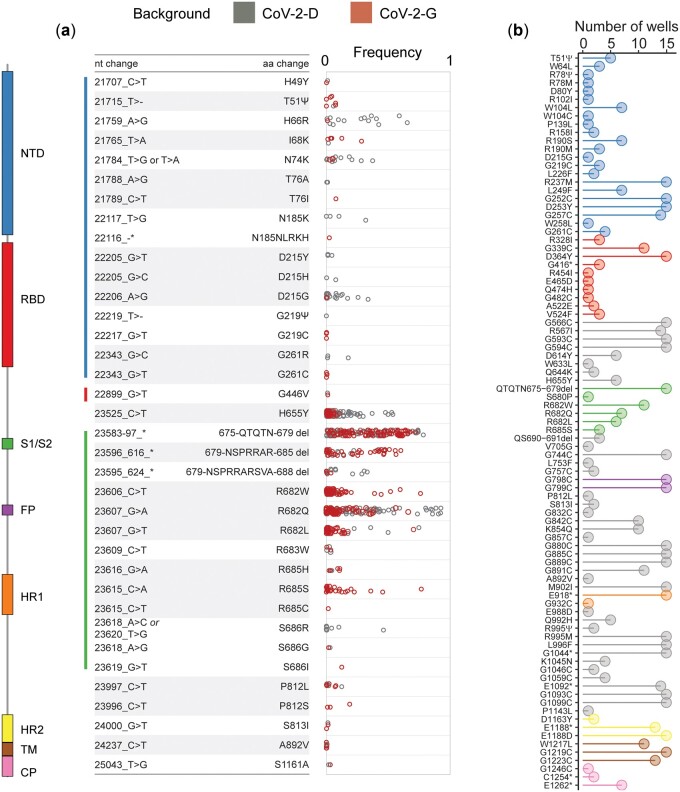
Convergent evolution in the Spike gene. (**a**) Amino acids of S targeted in both CoV-2-D and CoV-2-G backgrounds and their frequencies in each well (open circles). (**b**) Non-synonymous mutations on the spike detected in the populations where the mutators were observed (number of wells on the *X*-axis). The color annotation represents the N-terminal domain (NTD, 14–305), the receptor-binding domain (RBD, 319–541), the cleavage site (S1/S2, 669–688), the fusion peptide (FP, 788–806), the heptapeptide repeat sequences (HR1, 912–984 and HR2, 1163–1213), the TM domain (1213–1237) and cytoplasm domain (CP, 1237–1273)

The spike protein showed clear signs of adaptation during our evolution experiment, so we next focused on the specific sites under selection and compared them with the new spike variants that spread in the human population. We first quantified the level of convergence at the nucleotide level between CoV-2-D and CoV-2-G spike proteins. We note that convergence between the two backgrounds reflects true independent origin of the mutations, as they were propagated and processed for sequencing independently. In contrast, convergence within replicates of the same background could also result from some possible cross-contamination or from undetected standing variation. Considering the SNPs accumulated in each line, the average nucleotide diversity between backgrounds (6.7 ± 0.05) is greater than that within backgrounds (4.5 ± 0.06 and 5.6 ± 0.08 for CoV-2-D and CoV-2-G, respectively), suggesting some degree of genotype-dependent evolution (see Methodology). Nonetheless, significant convergence was observed at both nucleotide and amino acid levels. In the spike protein, 21 specific sites and 3 larger regions were hit independently in both backgrounds (non-synonymous changes shown in Fig. 6a, Supplementary Table S4), suggesting convergent adaptation since this would be extremely unlikely by chance ($p\left(x\geq 21\right)<10^{-5}$, hypergeometric test). We find high evolutionary convergence at the S1/S2 cleavage site: three distinct deletions (675-QTQTN-679 del; 679-NSPRRAR-685 del and 679-NSPRRARSVA-688) emerged multiple times in both backgrounds. Such changes have been previously shown to emerge rapidly in Vero cells and to be important for the virus cell tropism [39, 40]. On the other hand, the 678-TNSPRRARS-686 deletion, which should also trigger a similar functional effect, i.e. knock out of the furin cleavage site [41, 42], emerged multiple times, but was exclusive of CoV-2-D lines (*n* = 58). Apart from these deletions, other mutations in the S1/S2 region (e.g. Arginine 682, see Fig. 6) were also highly convergent, highlighting the adaptive potential of the cleavage site when evolving on Vero cells.

Some level of evolutionary convergence could also be found for the structural genes N, E and M suggesting that adaptation could also have occurred in these genes (Supplementary Table S5).

The inferred mutators in the CoV-2-G background also carry many mutations in the spike protein including in the receptor-binding domain (RBD; amino acid changes at positions 328, 339, 364, 416, 454, 465, 474, 479, 482, 522 and 524) and multi cleavage site regions (positions 798 and 799) (Fig. 6b). Several amino acid changes that occurred in our experiment were also observed in the natural population of SARS-CoV-2, which is not unexpected considering the massive volume of sequence data generated so far. Nonetheless, among these, we report the mutations H655Y (present in the variant of concern Gamma, lineage P.1, originated in Brazil, and Omicron, lineage BA.1), D215G (present in the variant of concern Beta, lineage B.1.351, first identified in South Africa) and D253G (found in lineage B.1.426, mostly detected in the USA) (Fig. 6) [43].

## CONCLUSIONS AND IMPLICATIONS

The SARS-CoV-2 beta-coronavirus, first observed in the Wuhan province of China [11], has infected millions of humans causing more than a 5 million toll of deaths (as of 2 November 2021; https://covid19.who.int/). Since it was first sequenced [44], the virus has been accumulating 0.44 substitutions per week at close to linear rate. Here, we followed the evolution in cells of two strains of SARS-CoV-2: one with the original spike protein (CoV-2-D) and one carrying the D614G mutation (CoV-2-G), a variant that emerged in the early phase of the pandemic and soon became prevalent [27]. We did not observe differences in their spontaneous mutation rates. Our estimate, for both backgrounds, is of the order of 0.1 per genome per infection cycle (between 1 and 5 × 10^−6^ nt^−1^ cycle^−1^), consistent with previous estimations in other coronaviruses [14]. New beneficial mutations did spread to high frequencies and considerable convergent evolution was detected between the CoV-2-D and CoV-2-D backgrounds. The two genomic backgrounds shared several *de novo* mutations, suggesting that the D614G mutation on the spike protein did not substantially alter the mutational path of the viral populations (Fig. 6).

Despite the similarities, we did observe a reduced accumulation of beneficial mutations in the CoV-2-G (particularly in its spike protein, see Fig. 3). Assuming that the D614G genotype is better adapted than its ancestor in Vero cells, this observation is consistent with the hypothesis that fitter genotypes adapt at a slower pace [45] (Fig. 1d).

After 15 days of propagation in Vero cells, we could also observe mutators emerging with a distinct mutation bias (G>T). This occurred only in the lines carrying the D614G mutation (CoV-2-G strain), but we cannot discard the null hypothesis that this happened in such genomic background simply by chance. The emergence of mutator phenotypes has not been documented in the human population, possibly because it is difficult to identify at low frequencies or simply because it is linked to our specific experimental conditions. Our data demonstrate that it is possible for SARS-CoV-2 to increase its mutation rate and survive, over the course of 15 propagation cycles in Vero cells. Given the caveats, this observation might be relevant for the development of strategies involving mutagen drugs [46, 47], especially if the drugs are being tested against SARS-CoV-2 grown in Vero cells.

Our study presents a number of limitations. Importantly, the experiments were conducted with Vero E6 monkey cells and need to be interpreted accordingly. Several studies (including ours) have shown that, propagating the SARS-CoV-2 virus in Vero cells can lead to rapid increase in frequencies of genetic variants with mutated furin cleavage site [39–42], demonstrating that the selective pressures in such environment do not reflect what happens during human infections [48]. Regarding the emergence of mutations, we did our best at excluding the effect of selection, but future studies should investigate how the spontaneous mutation rate changes when propagating SARS-CoV-2 on different host cells.

Despite the limitations, our study reports important information about the basic biology of SARS-CoV-2: we confirm its remarkable ability to adapt to new environments [49, 50], in particular via evolution of its spike protein, we provide an estimation of its mutation rate in cells and report the first spontaneous emergence of SARS-CoV-2 mutator phenotypes.

## ETHICAL STATEMENT

The Portuguese NIH is authorized by the Portuguese Authorities’ (General-Directorate of Health and the Authority for Working Conditions) to handle and propagate Risk Groups 2 and 3 microorganisms. All culture procedures were performed inside a Class III microbiological safety cabinet in a containment level 3 facility. This study is covered by the ethical approval issued by the Ethical Committee (‘Comissão de Ética para a Saúde’) of the Portuguese National Institute of Health.

## SUPPLEMENTARY DATA

Supplementary data are available at *EMPH* online.

## Supplementary Material

eoac010_Supplementary_Data
