# Supplementary material for: Mutation rate of SARS-CoV-2 and emergence of mutators during experimental evolution
Source: Evol Med Public Health. 2022 Mar 29;10(1):142–55. doi: 10.1093/emph/eoac010 (PMC8996265; doi:10.1093/emph/eoac010)
Supplement: eoac010_Supplementary_Data [file eoac010_supplementary_data.docx]

Supplementary Information for

Mutation rate of SARS-CoV-2 and emergence of mutators during experimental evolution

Massimo Amicone^1*^, Vítor Borges^2*^, Maria João Alves^3*^_,_ Joana Isidro^2*^_,_ Líbia Zé-Zé^3,4^, Sílvia Duarte^5^, Luís Vieira^5,6^, Raquel Guiomar^7^, João Paulo Gomes^2🖂^, Isabel Gordo^1🖂^

^1^Instituto Gulbenkian de Ciência, Oeiras, Portugal.

^2^Bioinformatics Unit, Department of Infectious Diseases, National Institute of Health Doutor Ricardo Jorge (INSA), Lisbon, Portugal.

^3^Centre for Vectors and Infectious Diseases Research, Department of Infectious Diseases, National Institute of Health Doutor Ricardo Jorge (INSA), Águas de Moura, Portugal.

^4^BioISI - Biosystems & Integrative Sciences Institute, Faculty of Sciences, University of Lisbon, Portugal.

^5^Innovation and Technology Unit, Department of Human Genetics, National Institute of Health Doutor Ricardo Jorge (INSA), Lisbon, Portugal.

^6^Centre for Toxicogenomics and Human Health (ToxOmics), Genetics, Oncology and Human Toxicology, Nova Medical School|Faculdade de Ciências Médicas, Universidade Nova de Lisboa, Lisbon, Portugal.

^7^National Reference Laboratory for Influenza and other Respiratory Viruses, Department of Infectious Diseases, National Institute of Health Doutor Ricardo Jorge (INSA), Lisbon, Portugal.

*These authors contributed equally to this work.

^🖂^e-mail: [igordo@igc.gulbenkian.pt](mailto:igordo@igc.gulbenkian.pt); [j.paulo.gomes@insa.min-saude.pt](mailto:j.paulo.gomes@insa.min-saude.pt)

**Supplementary Figures**


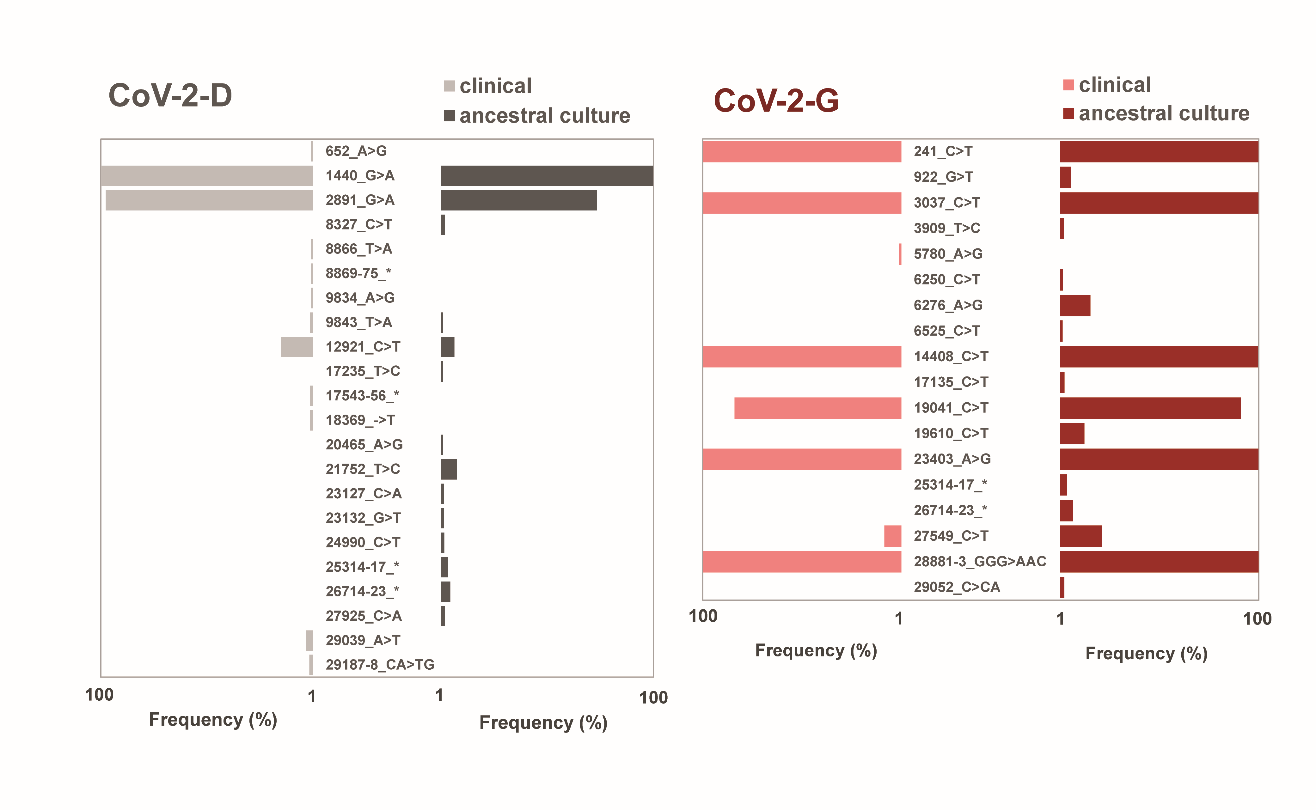


**Supplementary Fig. S1|** Mutations and their frequencies in the clinical isolates and their corresponding derived ancestors for the propagations. For the sake of figure simplicity, the complex mutations indicated in the figure with an asterisk correspond to: 8869-75_TTTGCCT>CAAACCA; 17543-56_TGTTCCTCGGAACT>AGTTCCGAGGAACA; 25314-17_GATC>TATG; 26714-23_TTTTGTGCTT>-GTTGTAC--.

**
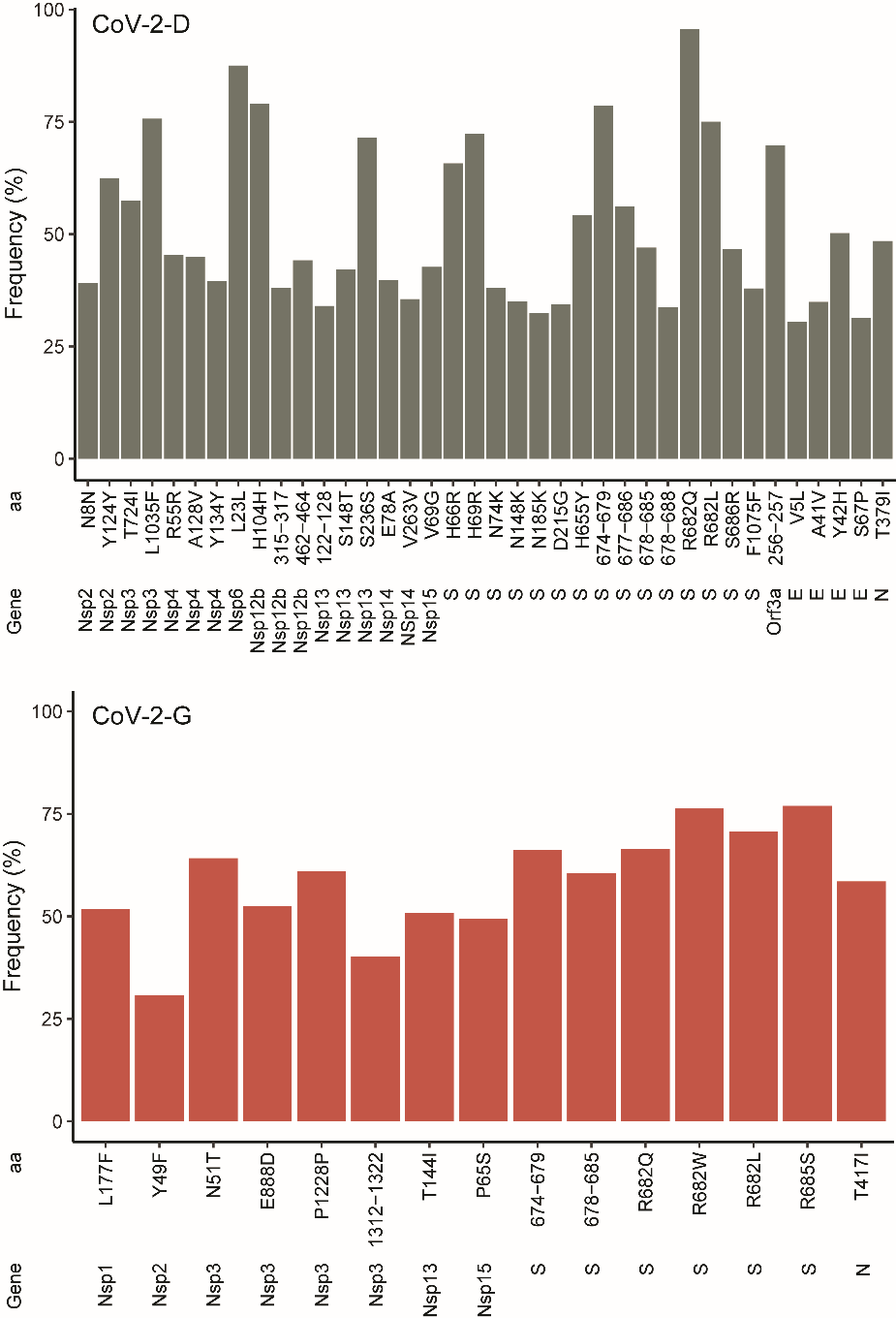
**

**Supplementary Fig. S2|** Mutations that reached frequency above 30% in each genomic background. The changes where deletions occur are indicated without letters (e.g. 674-679 corresponds to a deletion of YQTQTN to Y, see **Supplementary Table 1** for detailed annotation of all mutations).


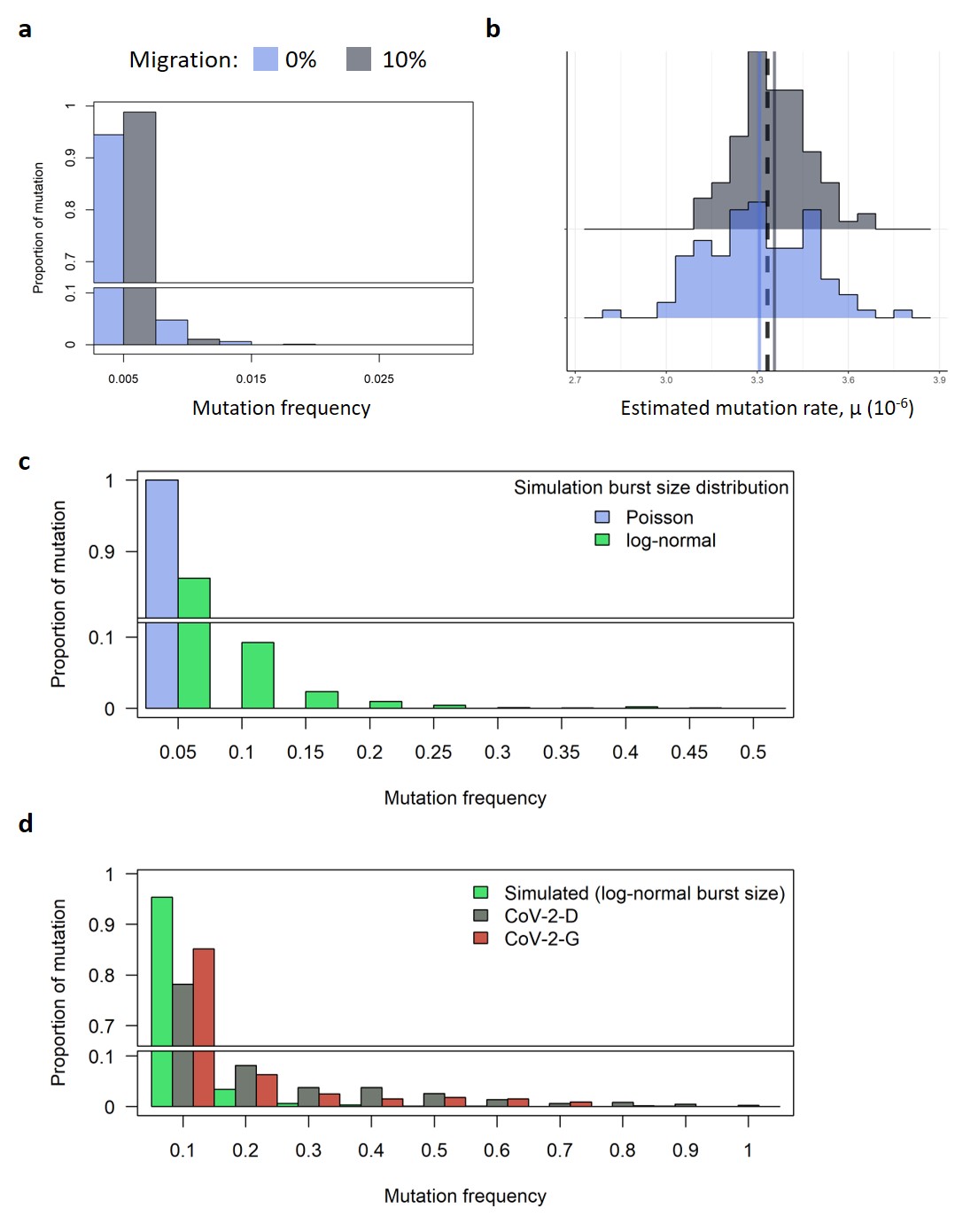


**Supplementary Fig. S3| a,** the neutral site frequency spectrum expected with or without 10% migration (cross contamination between wells) at each infection cycle. **b,** No effect of 10% migration on the estimation of mutation rate. The dashed line represents the simulated *μ* while the continuous lines represent the average of the estimated μ with or without migration. **c,** Comparison between the site frequency spectrum obtained with Poisson (as in **Fig. 3**) or log-normal distributed viral burst size. Both distributions have mean=1000. **d,** Equivalent to **Fig. 3**, with heavy-tail distributed burst size, instead.


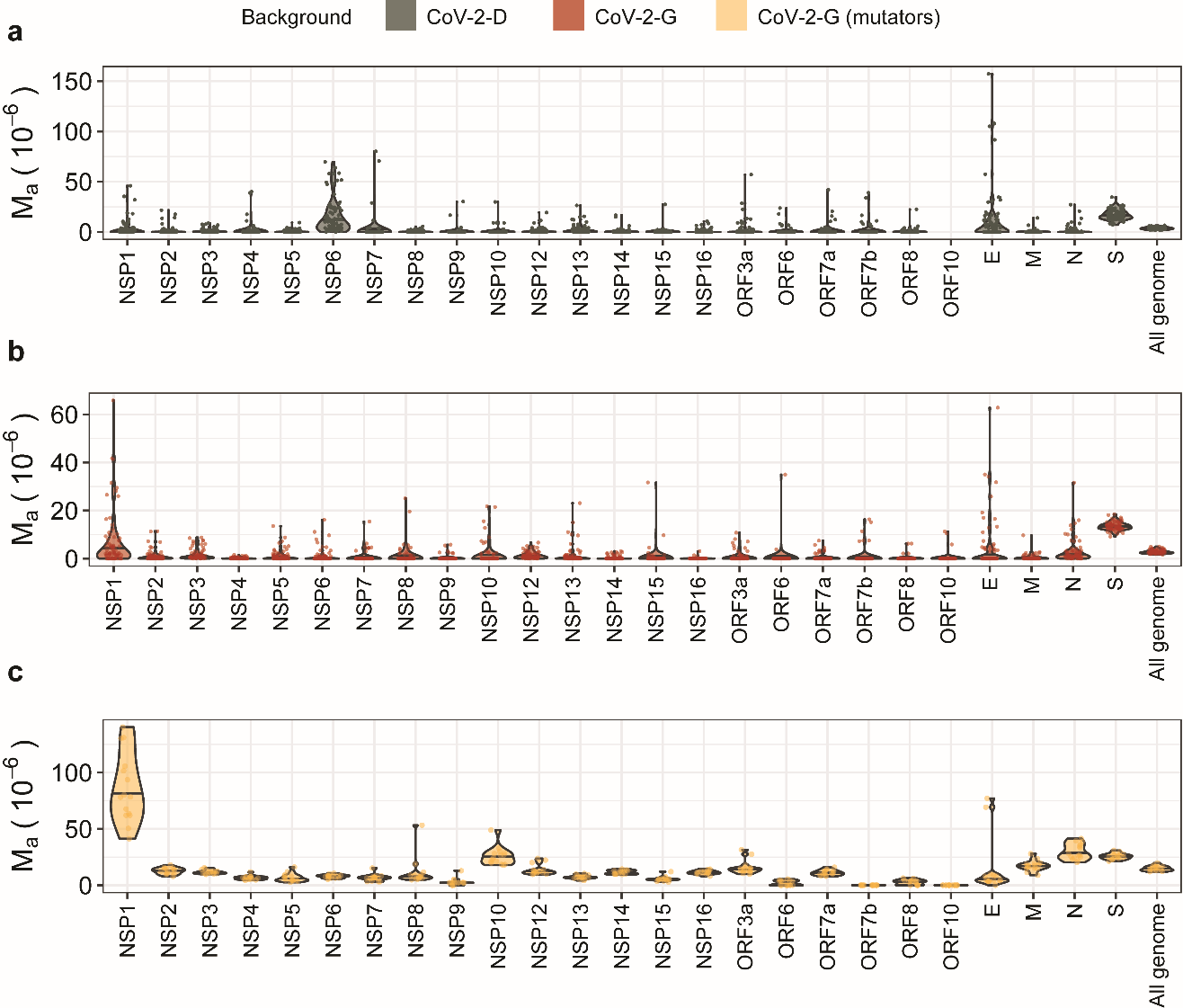


**Supplementary Fig. S4|** Heterogeneity of mutation accumulation across the genome of SARS-CoV-2. **a-c,** Per-base mutation accumulation (*M_a_*) computed for each gene and for the entire genome in the CoV-2-D (n=96), CoV-2-G (non-mutators, n=79) and CoV-2-G (mutators, n=15) backgrounds, respectively.


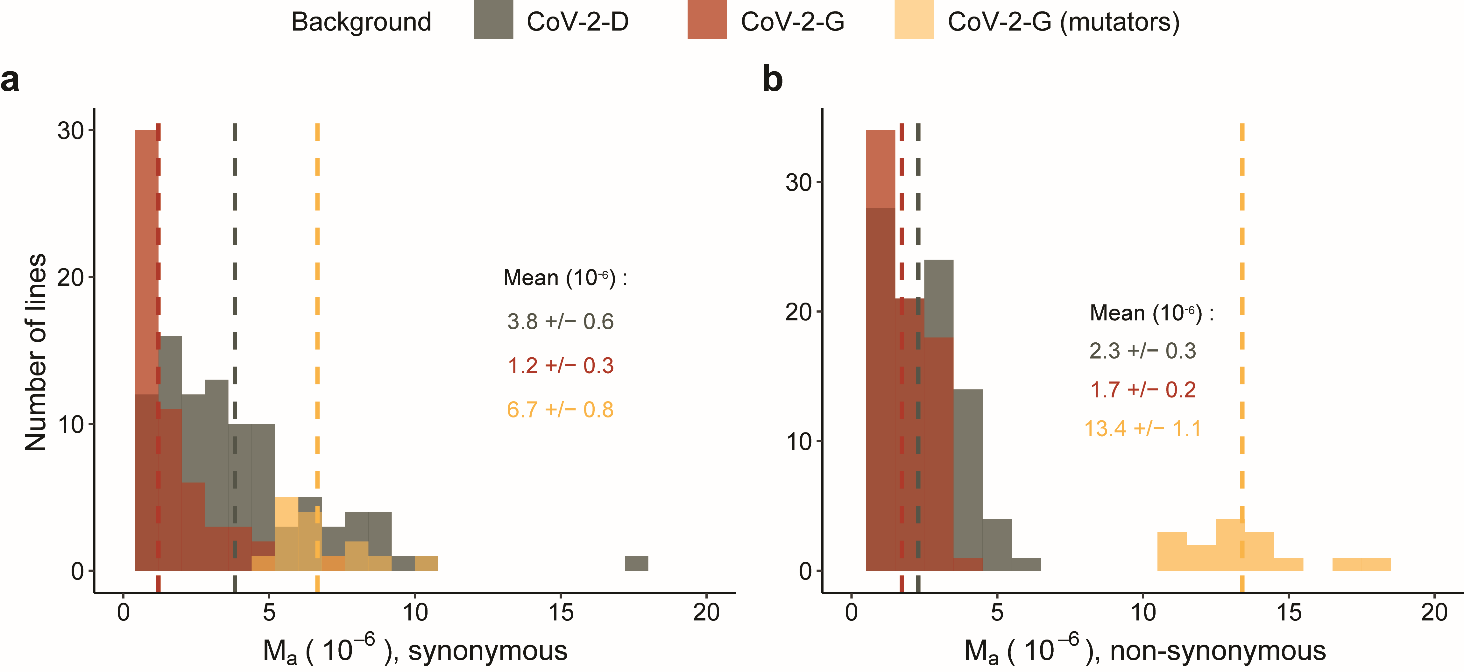


**Supplementary Fig. S5| a-b,** Per-base per-cycle mutation accumulation (*M_a_*) of synonymous (**panel a**) and non-synonymous changes (**panel b**). *M_a_* was calculated by summing the observed mutation frequencies as: $M_{a}=\frac{\sum f}{P*G}$, where *P* is the number of passages (*P*=15) and G is the number of synonymous or non-synonymous sites in the genome of SARS-CoV-2 (G=6418 and 22846, respectively). The means of each group are presented by vertical dashed lines and reported in the figure (+/- 2SEM).


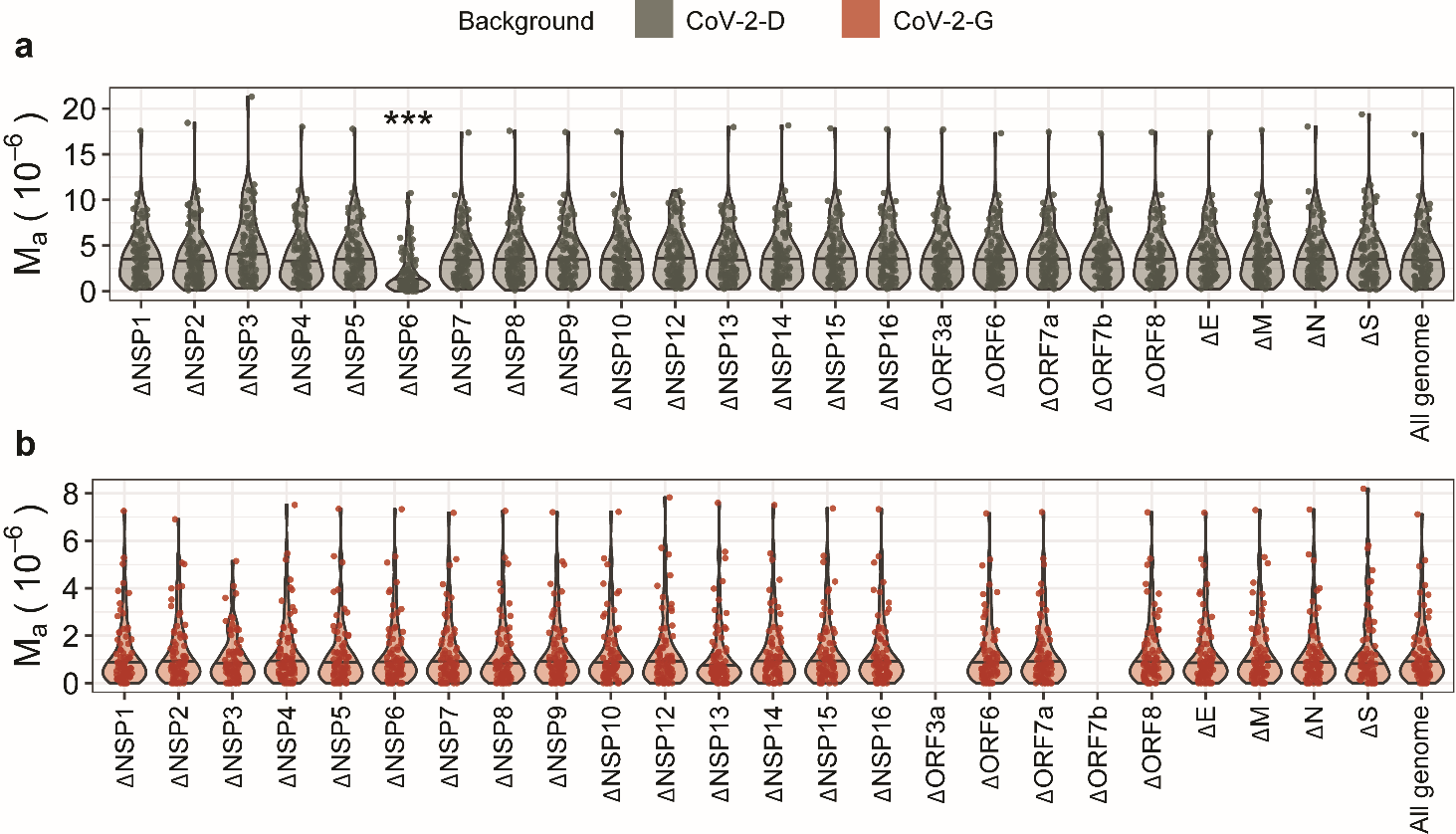


**Supplementary Fig. S6| a-b,** Outlier analysis using synonymous mutations only (notice that **Fig. 4b** in the main text is instead with all the mutations). The Nsp6 gene in CoV-2-D background is responsible for the overestimation of *M_a_* and should be removed for a more accurate estimation of the spontaneous mutation rate. Here, *M_a_* was computed from the synonymous mutations throughout the entire genome or by excluding each gene on at a time (e.g. ΔS). The stars indicate the cases where removing the gene leads to an estimation of *M_a_* significantly different from the entire genome (non-parametric Wilcoxon test, p-value < 0.05 (*), 0.01 (**) or 0.001 (***), after Benjamin-Hochberg correction).


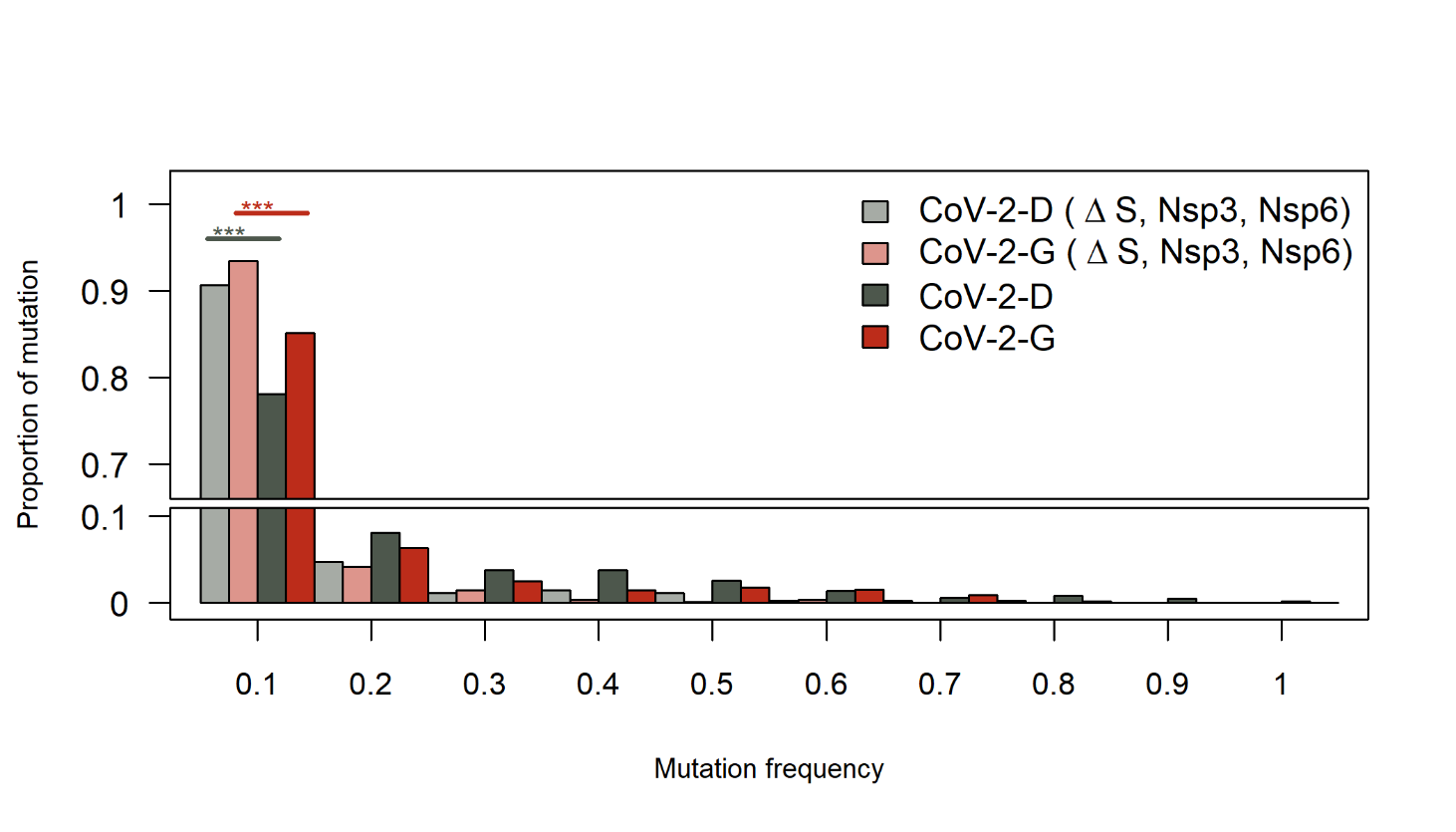


**Supplementary Fig. S7| Site frequency spectrum with and without the S, Nsp3 and Nsp6 genes.** Proportion of mutations with a given frequency after 15 cycles of propagation in the CoV-2-D and CoV-2-G genetic background, with (dark colors) or without (light colors) the S, Nsp3 and Nsp6 genes, which have shown signs of selection and/or a different accumulation of mutations. The tail of the distributions is reduced after removing those genes, indicating that, at least partially, selection had caused the high frequency mutations (see **Fig. 3**). We tested the tail reduction by comparing the proportion of mutations whose frequency is below 0.1, with and without the S, Nsp3 and Nsp6 genes. Stars indicate the level of significance (p-value <0.001 after two-proportions z-test and Benjamin-Hochberg correction).


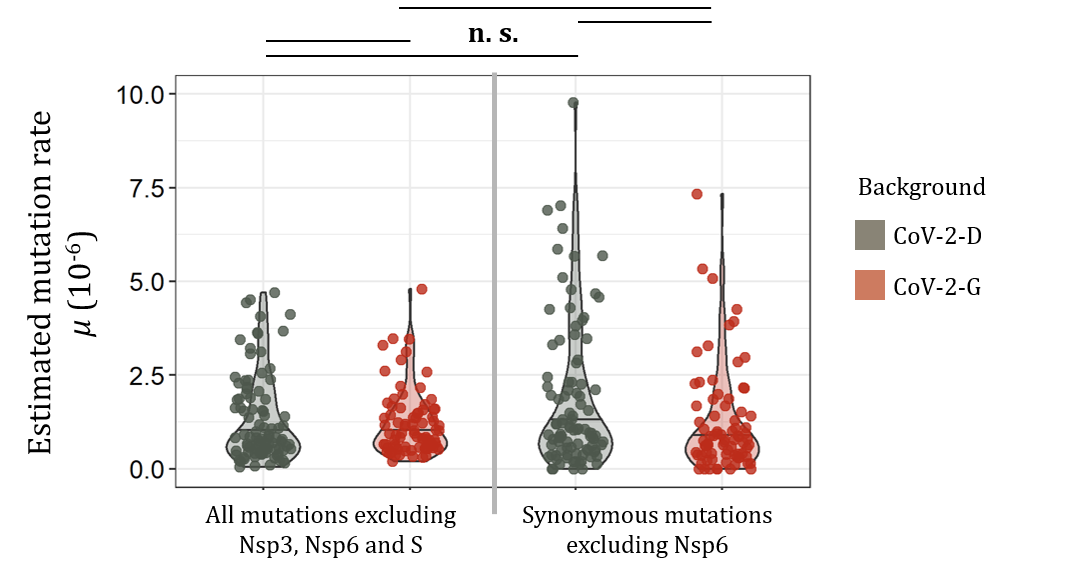


**Supplementary Fig. S8| Comparing estimations of mutation rate.** Estimation of SARS-CoV-2 mutation rate excluding the genes with signals of selection (Nsp3, Nsp6 and S) or considering synonymous mutations only (and excluding the outlier gene Nsp6). The estimations are equivalent between the CoV-2-D and CoV-2-G backgrounds and between the two approaches (non-parametric Wilcoxon test, p-value > 0.05, n. s.).


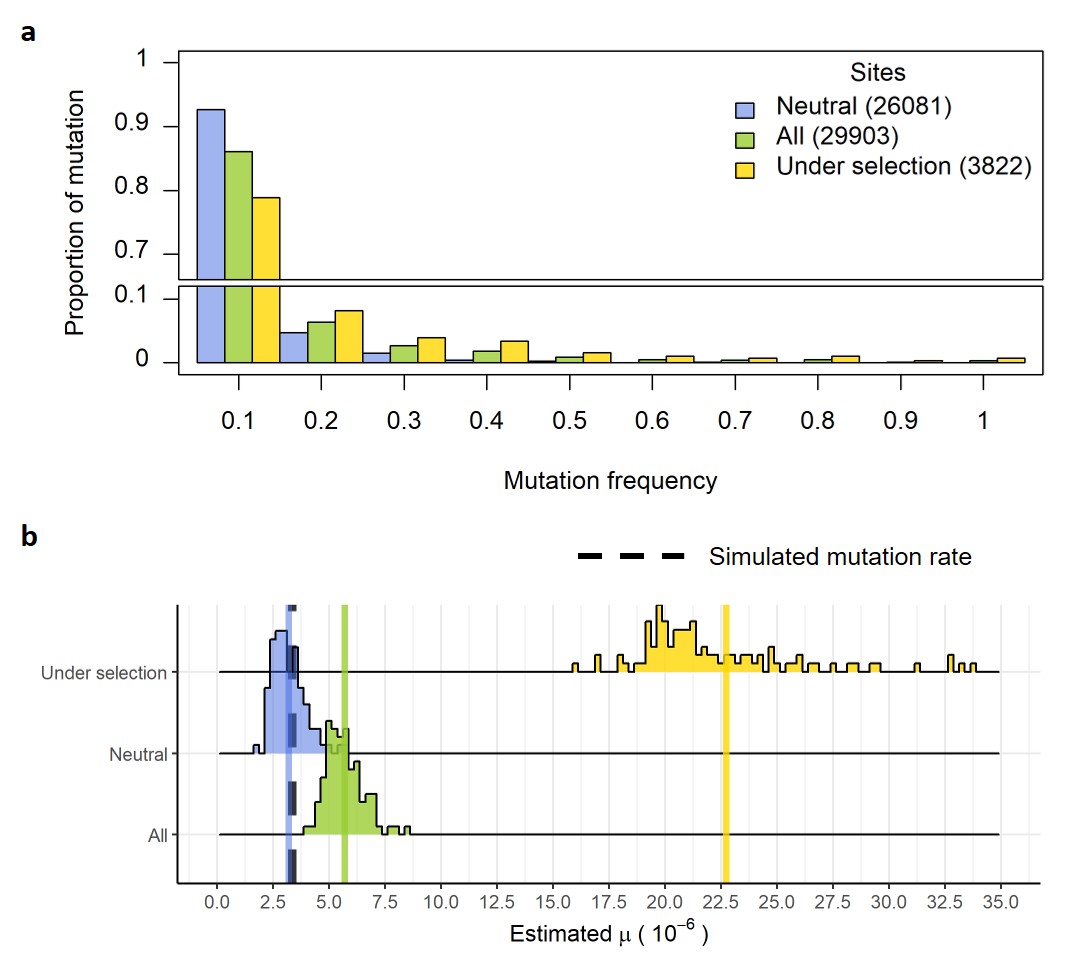


**Supplementary Fig. S9| Simulating the effect of hitchhiking.** Site frequency spectra obtained via simulating, 100 independent times, the evolution of a genome where 3822 sites (like the spike protein) are under selection ($s\sim Normal(\mu=0, \sigma=0.5)$) and the remaining 26081 are neutral. Neutral sites can reach frequencies larger than 0.1 by hitchhiking. **b,** Effects on the estimation of mutation rate. Selection acting on a given region (e.g. the spike protein) leads to an overestimation of mutation rate when this is computed within such region (yellow), within the entire genome (green), but does not affect the mutation rate computed on the neutral sites (blue). The true simulated mutation rate (∼3.3x10^-6^) is shown by the dashed black line.


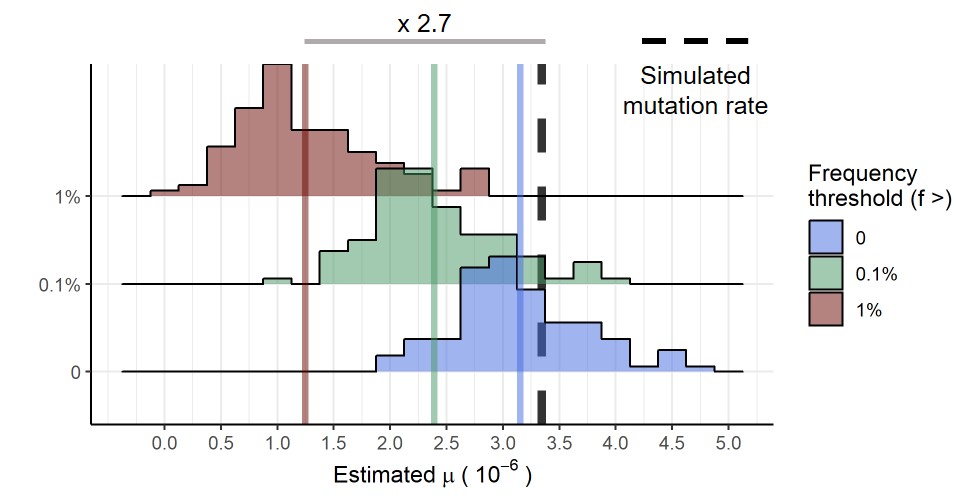


**Supplementary Fig. S10| Simulating the effect of different frequency thresholds.** Estimation of mutation rate from simulated neutral sites (blue distribution of **Fig. S9**), assuming different thresholds for mutation frequencies. To minimize sequencing errors, in our experiment we considered mutations above 1% frequency and according to the simulations this could lead to underestimate the real mutation rate by a factor of 2.7 (compare red distribution with the dashed line). The true simulated mutation rate (∼3.3x10^-6^) is shown by the dashed black line.

**Supplementary Tables**

Supplementary Tables 1 to 5 are uploaded as Excel files.

**Supplementary Table 1| Mutation summary and accession numbers.** List of all detected mutations and their distribution across clinical, ancestral cultures and end-point cultured lines (15th passage). MNP = multi-nucleotide polymorphism; complex = mutation event comprising SNP and indels; * = stop codon; fs = frameshift. Sheet2 contains the European Nucleotide Archive (ENA) accession numbers for the read sequencing data.

**Supplementary Table 2| Mutator-specific mutations on the Nsp12 gene.** List of all mutations in the Nsp12 gene, unique to the mutator lines that emerged in the CoV-2-G background.

**Supplementary Table 3| Mutator-specific mutations on the Nsp14 gene.** List of all mutations in the Nsp14 gene, unique to the mutator lines that emerged in the CoV-2-G background.

**Supplementary Table 4| Convergent mutations in the spike protein.** List of all mutations in the spike protein that converged at the nucleotide level between the evolved lines CoV-2-D and Cov-2-G.

**Supplementary Table 5| Convergent mutations in the N, M and E proteins.** List of all mutations in the N, M and E proteins, that converged at the nucleotide level between the evolved lines CoV-2-D and Cov-2-G.
